# Gliptins and Cardiovascular Outcomes: A Comparative and Critical Analysis after TECOS

**DOI:** 10.1155/2016/1643496

**Published:** 2015-11-15

**Authors:** Samit Ghosal, Binayak Sinha

**Affiliations:** ^1^Nightingale Hospital, 11 Shakespeare Sarani, Kolkata, India; ^2^AMRI Hospitals, JC-16/17, Salt Lake City, Kolkata 700091, India

## Abstract

The issue related to macrovascular outcomes and intensive glycemic control was hotly debated after the publication of landmark trials like ACCORD, ADVANCE, and VADT. The only benefits seem to come from intervening early on in the disease process as indicated by the 10-year UKPDS follow-up. To complicate matters USFDA made it mandatory for modern drugs to conduct cardiovascular safety trials in high-risk populations after the 2008 rosiglitazone scare. This led to all the modern group of drugs designing cardiovascular safety trials (gliptins, GLP-1 agonists, and SGLT-2 inhibitors) to meet USFDA regulatory requirements. We saw publication of the first 2 randomized trials with gliptins published a year and a half back. On the face value SAVOR TIMI and EXAMINE satisfied the primary composite CV end-points. However, issues related to significant increase in heart failure and all-cause 7-day on-treatment mortality created a lot of confusion. FDA reanalysis of these data (especially SAVOR) raises a lot of doubts as far as CV safety of these groups of drugs was concerned. Hence, all eyes were on TECOS, which was published this year. We take a microscopic look at these trials trying to understand where we stand as from now on this issue.

## 1. Introduction

The adverse impact of oral antihyperglycemic agent on the cardiovascular system came into focus as early as 1971 with UGDP program [1]. The drug in question was a sulfonylurea. However, with the advent of second-generation sulfonylureas and nonusage of first-generation ones this controversy was put to rest (without proper investigation).

In early 2000 it was glitazones which came into the headlines with issues related to fluid accumulation (pedal edema and macular edema) [2]. Glitazone group of drugs were contraindicated in patients with NYHA Classes III and IV heart failure [3].

There was another development in 2005 which brought the issue of secondary end-points into focus. It was the PROactive study [4]. The results of this study were eagerly awaited, as this was the first time type 2 diabetic individuals with high CV risk were exposed to a CV outcomes trial. All the surrogate CV markers positively influenced by pioglitazone were put to test [5]. The primary end-points put to test over an average of 34.5-month follow-up were time to first death, nonfatal MI, stroke, acute coronary syndrome, major leg amputation, coronary revascularization, and leg revascularization.

The key findings are summarized in Table 1.

The primary end-points in this study failed to achieve statistical significance in spite of a significant glycemic difference between the two arms (−0.8% pioglitazone versus −0.3% placebo; *P* < 0.0001) [4]. It was a huge disappointment. However, it was at this point that we saw the secondary end-points gaining a lot of significance. Instead of being a negative trial PROactive was suddenly a positive trial. However, one of the secondary end-points, that is, heart failure, was grossly underhighlighted. There were significantly higher event rates related to heart failure in the pioglitazone arm (Table 2).

The main blow to modern drugs seeking regulatory approval came from a meta-analysis involving rosiglitazone. Nissen and Wolski presented data analyzing 42 randomized trials with control group and of 24-week duration [6]. The baseline characteristics are summarized in Table 3.

There was a statistically significant 43% increase in myocardial infarction with a trend towards increased death from cardiovascular causes in the rosiglitazone arm compared to active comparators and placebo [6] (Table 4).

The very next year (2008) USFDA came up with “Guidance for Industry” [7]. The new guidance stressed on the sponsors recruiting an independent committee looking into the cardiovascular end-points in the phases II and III study programs. The primary and secondary end-points along with the methods employed for statistical analysis should be clearly mentioned. A two-sided 95% confidence interval for each assessment of risk ratio was formulated. The cut-off values set as mandatory requirements are presented in Table 5.

It was pointed out, however, that individuals recruited in phases II and III programs would mostly be younger with a short duration of diabetes (not the ideal population to assess CV outcomes). Hence, it was mandatory to conduct a CV safety outcomes trial even when there were no adverse signals in the phases II and III programs especially if the CV event rates were low [7].

The modern antidiabetic medications as a result of the abovementioned developments were exposed to a new set of laws.

Keeping in mind the USFDA CV safety requirements and the importance of secondary end-points especially heart failure, let us take an in-depth look at the recently published CV outcomes data with gliptins in focus.

## 2. The Gliptin Era

In mid-2000 we got a new group of antihyperglycemic agents (DPP 4 inhibitors or gliptins) for the management of type 2 diabetes. The advantages associated with this group were lack of hypoglycemia and weight neutrality [8]. Added to metformin, gliptins have an equivalent HBA1C reducing ability compared to sulfonylurea [9]. Subsequent data pointed at additional benefits associated with gliptins as far as CV surrogates were concerned [10]. Sitagliptin was associated with reduction in postmeal triglyceride rich apo-B levels [11]. Similarly, saxagliptin was associated with reduction in blood pressure in animal models [12].

All these positive effects pointed at the possibility of CV outcomes benefit with gliptins compared to placebo or other active oral comparators.

## 3. The Polled Phase 2/3 Saxagliptin Data

USFDA analyzed the pooled phases 2b and 3 saxagliptin data, which included a total of 8 studies and came out with their report in 2009 [13]. The primary MACE was the focus of attention. The major obstacle in the way of analyzing the data was deriving an accurate definition and terminology for the individual MACE events. Apart from the sponsor's definition of MACE, two more definitions were included in this analysis. The first was “Broad SMQ MACE” and the second “Custom MACE” [13]. SMQ stands for standardized MedDRA (Medical Dictionary for Regulatory Activities) queries. The terminologies used to define a disease entity were in accordance with a standardized definition. Custom MACE was a subset of “Broad SMQ MACE” which is more specific as far as defining the end-points was concerned supervised by 3 FDA reviewers [13]. Saxagliptin arm fared very well as far as the primary end-points were concerned (Table 6).

The MACE data indicates that both the custom and sponsor MACE achieved the FDA recommended cut-off value of less than 1.3 [13]. The ST custom MACE as a matter of fact reached statistical significance. The SMQ MACE was diluted with the use of CPK as one of the preferred terms thereby increasing the number of events and the upper limit of the two-sided confidence interval crossing 1.3 [13].

In a similar analysis of data with linagliptin the sponsor MACE (0.36 [95% CI 0.17–0.78]) as well as FDA Custom MACE (0.34 [95% CI 0.15–0.75]) met the FDA cut-off criteria [14].

However, the number of CV events in the saxagliptin pooled data custom MACE was 40 and that in the linagliptin pooled data was 11 [13, 14]. This was an extremely small number to come to a definitive conclusion as far as CV safety was concerned. Hence, it was mandatory to undergo a dedicated CV safety trial. All the gliptins took to dedicated CV safety trials except vildagliptin.

## 4. Gliptins: CV Safety Data (SAVOR TIMI-53, EXAMINE, and TECOS)

SAVOR TIMI-53 (saxagliptin) and EXAMINE (alogliptin) were published in 2008 whereas TECOS (sitagliptin) was published in 2015. Let us take a look at the salient CV safety issues highlighted in these three landmark trials.

### 4.1. SAVOR TIMI-53 (Saxagliptin)

The aim of this study was to analyze primary efficacy (superiority), primary safety (noninferiority), and secondary safety issues in an intention to treat (ITT) population. It was estimated from the annual primary CV event rate with placebo (2.8%) that the study should recruit 12,000 patients and run the trial for 2 years with 3-year follow-up (total of 5 years duration) to get hold of 1,040 CV events [15]. This would enable the investigators to generate adequate power to test the noninferiority hypothesis. However, after 10 months after recruitment the investigators realized that the numbers recruited would not yield the anticipated event rates and additional subjects (with established CVD) were recruited to increase the study population number to 16,500 [16].

This study differed significantly from the other CV trials on the following points:

- Baseline HBA1C was over a broader range (>6.5% to ≤12.0%) with a mean value of 8.0% (±1.4%) versus TECOS (HBA1C range: 6.5–8.0%) [15, 17].
- There were patients without established CVD recruited in this trial (primary CV prevention cohort). Recruitment of this population was restricted to 25% of the whole study population [15].

The results were published in October 2014. The prominent primary end-point results were as follows:

(i) Primary efficacy end-point (superiority): HR 1.0 (0.89–1.12); *P* = 0.99. It failed the superiority test [16].
(ii) Primary safety end-point (noninferiority): *P* < 0.001 (passed the noninferiority test) [16].

The areas of concern from CV perspective are as follows:

(i) Although designed as a glycemic equipoise study, the end results revealed a statistically significant difference between the two arms as far as fasting plasma glucose and HBA1C were concerned. There were significantly more episodes of both minor (*P* = 0.002) and major (*P* = 0.047) hypoglycemic episodes in the saxagliptin arm compared to placebo [16].
(ii) There was a statistically significant 27% increased rate for hospitalization due to heart failure (HR 1.27 [1.07–1.51]; *P* = 0.007) [16].
 Although a distinctive mechanism leading to heart failure was not obvious with gliptins, several groups speculated whether it was the impact of DPP-4 inhibitors on substrates like Neuropeptide-Y (NP-Y) and Substance P (SP) that could lead to this effect [18]. Others speculated whether the additional patients with established CVD recruited later in the trial could lead to this effect. Till date, we do not have any answer to this phenomenon. The next logical step would be to wait for another CV safety trial.

### 4.2. EXAMINE (Alogliptin)

EXAMINE study group investigators recruited patients exclusively with established ACS. Since patients with established ASC were included in this trial, the placebo annual primary MACE was estimated at 3.5% [19]. As a result, recruiting a smaller population (5,400) followed up for approximately 40 months would generate adequate enough power to fulfill the HR cut-off [19].

At the end of the trial there was a statistically significant 0.36% greater HBA1C reduction (*P* < 0.001) with alogliptin compared to placebo (usual care) [20]. Once again the glycemic equipoise hypothesis was not satisfied.

The alogliptin arm achieved the primary end-point (death from cardiovascular causes, nonfatal myocardial infarction, or nonfatal stroke) noninferiority (HR 0.96; upper boundary of CI 1.16) [20].

With the adverse impact of saxagliptin in SAVOR TIMI-53 on hospitalization for heart failure as well as increased hypoglycemic episodes, all eyes were on EXAMINE as far as these parameters were concerned:

- At baseline 24.2% of the recruited patient population had congestive heart failure [19]. However, NYHA Class IV was an exclusion criterion. EXAMINE data revealed that there was no significant trend as far as hospitalization due to heart failure as a first event was concerned (HR: 1.07; 95% CI: 0.79–1.46) [20].
- There were no significant differences between the two groups as far as the rates of hypoglycemic events were concerned.

On the one hand, the results of EXAMINE were a bit reassuring while on the other hand the absence of a clear cut answer as far as hospitalization from heart failure was concerned was discomforting. In an editorial on the post hoc analysis of heart failure, it was pointed out that there was a statistically nonsignificant increased risk for hospitalization due to heart failure (HR 1.19 CI: 0.89–1.57) [21]. As a matter of fact those without a background history of heart failure had a significant risk of developing it while being on alogliptin (HR 1.76; 95% CI: 1.07–2.90) [21].

Hence, all eyes were on the upcoming CV safety trial with gliptins and the one closest to publication after SAVOR TIMI-53 and EXAMINE was TECOS.

However, at this point FDA took note of the adverse signals emerging from these trials and conducted an analysis of their own.

## 5. FDA Analysis of SAVOR TIMI-53 and EXAMINE

### 5.1. Reanalysis of SAVOR TIMI-53

Four prominent issues were up for discussion [22]:

- The issue related to heart failure.
- Comments that were sought on all-cause mortality.
- Issues related to renal safety.
- Any additional safety issues.

We will focus on the CV safety related issues only in this review.

While reanalyzing the sponsors existing data, FDA introduced the term mITT, which differed from ITT (intention to treat) in terms of the fact that the subject must have received the drug in question even if it is for a day. This “on-treatment” analysis was further subdivided into +7 days and +30 days' analysis (i.e., events occurring 7 days and 30 days after receiving the last dose of saxagliptin or placebo): the time-to-event analysis [22].

Another important point to remember at this point is that all-cause mortality was a stand-alone secondary end-point whereas hospitalization for heart failure was one of multiple secondary end-points (MACE+).

### 5.2. The Reanalysis Results

#### 5.2.1. Primary MACE

Both the ITT (HR: 1.00; 95% CI: 0.89–1.12) and the mITT (HR: 1.00; 95% CI: 0.89–1.12) for primary MACE satisfied the FDA requirements (upper limit of CI <1.3) [22].

The individual components of primary MACE did not differ either.

#### 5.2.2. Secondary End-Points (MACE+)

The prespecified ITT secondary end-point composite (MACE+) had a hazard ration of 1.02 with the upper boundary of CI at 1.11 [22].

Amongst the individual secondary end-points, it was hospitalization due to heart failure, which was pointing the wrong way. The problem FDA identified was that this was not a prespecified stand-alone secondary end-point. Since this end-point analysis ended up with a statistically significant difference, FDA went ahead and reanalyzed the data. Although those in the secondary end-point arm could continue on in the trail, those experiencing a primary event were not followed up any more. This results in crunching of the high-risk population base as we go on. Hence, FDA designed a new analytical process whereby subjects with heart failure or other high-risk composites were included in the analysis (e.g., hHF or primary MACE; hHF or CV death) [22]. On-study ITT population was analyzed.

Of the 3 end-points analyzed (hHF or primary MACE, hHF or CV death, and hHF or all-cause death) hHF or CV death (HR: 1.14; 95% CI: 1.00–1.30) and hHF or all-cause death (HR: 1.16; CI: 1.03–1.30) had the lower boundary of CI at or above 1 [22]. Hence, the issue of hHF remained significant even in the reanalysis performed.

It was thought that the detrimental by-products of DPP-4 inhibition Neuropeptide-Y (NP-Y) and Substance P (SP) clubbed with ACE inhibition could lead to increased sympathetic activity and heart failure. However, the data indicated a higher and significant hazards ratio (HR 1.42; 95% CI: 1.09–1.88) for time to first hospitalization due to heart failure in those not on ACE inhibitors compared to those who were on it (HR 1.18; 95% CI: 0.94–1.48) [22]. Hence, the DPP-4 inhibition induced production of adverse by-products (NP-Y and SP) could not explain this end-point.

Although a lot of effort was spent analyzing why there was an increase in hospitalization due to heart failure, none of the hypothesis generated could explain this phenomenon.

*All-Cause Mortality*. This was a stand-alone secondary end-point.

This was another area of concern. Although the prespecified IIT analysis did not show any trend towards an increase in all-cause mortality (HR: 1.11; 95% CI: 0.96–1.27), the mITT analysis pointed at a statistically significant increase in 7-day death (HR: 1.23; 95% CI: 1.02–1.48) (Table 7) [22]. Once again we come across a number of speculations attempting to explain the increased rates of all-cause mortality. A sizable 25% of patients in SAVOR TIMI had a baseline HBA1C below 7%. This clubbed with statistically increased rates of hypoglycemia in the saxagliptin arm was touted as one of the reasons. However, the FDA analysis on the same and other points (heart failure, increased rates of arrhythmias, etc.) could not arrive at a definitive conclusion [22]. Nevertheless, the FDA document did not consider this increased risk as a pattern happening by chance.

### 5.3. Reanalysis of EXAMINE Trial

All eyes were on the reanalyzed heart failure and all-cause mortality data as the primary end-point was met.

### 5.4. Heart Failure Reanalysis Data

The same analytical method was employed as in the case of SAVOR TIMI. A composite of MACE or heart failure was looked into and the data was encouraging (HR: 0.982; 95% CI: 0.848–1.138) [23]. There were no signals of increased first hospitalization from heart failure. However, it should be mentioned at this point once again that EXAMINE trial excluded patients with NYHA Class IV and had the majority of individuals in the NYHA Class II category [23].

### 5.5. Mortality Signals

Patients recruited from US and Canada had a higher hazard ratio of MACE (HR: 1.28; 95% CI: 0.89–1.84) (Table 7) [23]. Although there were differences as far as baseline characteristics were concerned (longer duration of diabetes, more smokers, etc.), these factors could not explain why there was an increased mortality trend.

Increased hazard ratio for MACE was also observed in those with longer duration of diabetes (>10 years duration), moderate-to-severe renal disease, biguanide nonusers, and insulin users (Table 7) [23].

It was reassuring to note that all-cause mortality was not increased in this trial (HR: 0.876; 95% CI: 0.705–1.089) [23].

### 5.6. Post-FDA Reanalysis of Data in 2015

There were mixed reactions. Some schools of thought felt that the issue of heart failure was not replicated and hence there was some reassurance on this issue. On the other hand, the critics pointed out that there were inherent differences between the trials and hence it was not possible to come to a definite conclusion.

Hence, all eyes were on TECOS.

### 5.7. The TECOS Data

*Baseline Characteristics in Brief*. TECOS recruited patients with a compact HBA1C range (6.5–8.0%) and established cardiovascular disease. Patients with eGFR <30 mL/min were excluded from the trial [24].

The placebo annual rate of CV events was estimated at 2.5–3.0% for primary composite events. Hence, it was estimated that recruiting 14,1000 patients would result in approximately 611 CV events over 6 years [24].

### 5.8. The Results

Once again we are looking at a glycemic equipoise study. However, the end-of-study HBA1C difference of 0.29% reached statistical significance (95% CI: −0.32 to −0.27; *P* < 0.0001) [17].

*Primary CV End-Points*. Both PP (HR: 0.98; 95% CI: 0.88–1.09) and ITT (HR: 0.98; 95% CI: 0.88–1.09) analysis echoed the findings from SAVOR TIMI and EXAMINE [16, 17, 20]. Sitagliptin satisfied the FDA upper bound CI of <1.30 as far as primary CV outcomes were concerned. However, sitagliptin could not meet the superiority criteria (HR: 0.99; 95% CI: 0.89–1.10) [17].

### 5.9. The Areas under Focus

All eyes were focused on the heart failure and mortality issues.

The ITT analysis of hospitalization due to heart failure did not reveal any concerns (HR: 1.00; 95% CI: 0.83–1.20; *P* = 0.98). What was interesting to note was that unlike the previous studies TECOS utilized the FDA pattern and analyzed hHF or cardiovascular death composite for the ITT population. Once again the results were reassuring (HR: 1.02; 95% CI: 0.90–1.15; *P* = 0.74) [17].

All-cause mortality was also not adversely affected (HR: 1.01; 95% CI: 0.90–1.14) [17]. What was reassuring to note was that there were no adverse MACE signals as far as differing patient population, duration of diabetes, baseline HBA1C, baseline nonuse of biguanides, insulin usage, or use of ACE inhibitors were concerned [17]. This was in direct contrast to the findings from EXAMINE trial.

## 6. Conclusion

The issue of adverse impact of oral hypoglycemic agents and adverse CV signals can be traced back to UGDP in 1971 [1]. Similar trends were also observed in UKPDS 34 when adding metformin to sulfonylurea resulted in an increase in diabetes-related deaths and all-cause mortality [25].

However, these data were not given a lot of attention as there were issues related to trial design, analysis, and interpretation.

The modern trials on the contrary are well designed and analyzed. There is no running away from the fact that dedicated CV safety trials will be required for all the modern drugs to find a definitive place in treatment algorithm. With this background all the three randomized trials (SAVOR TIMI, EXAMINE, and TECOS) reassure us on the CV safety of DPP-4 inhibitors from the primary end-point perspective. What keeps the debate going are the issues related to increased hypoglycemia risk, hHF, and increased all-cause mortality in SAVOR TIMI and increased MACE risk in certain population in EXAMINE. TECOS was the picture-perfect trial pulling along the other drugs in this group towards the positive side and ruling out the fear of class effect as far as the adverse events were concerned.

However, it would be premature to say that the issue has been settled once and for all.

There are a couple of interesting studies lined up either to simplify the contentious issues or to complicate matters even further. CARMELINA with linagliptin would add on to the increasing experience with usage of DPP-4 inhibitors in high CVD risk diabetic population [26]. Another data would probably help put a lot of unanswered questions in perspective.

However, things might get a bit complicated with CAROLINA [27]. This study recruited patients with newly diagnosed treatment naïve diabetes or early on in the disease process with high CV risks profile. Patients were randomized to either glimepiride or linagliptin and would be followed up for 5 years. This would be the first in kind head-to-head comparison between a sulfonylurea and a gliptin on high CV risk patients. The duration of this trial is too short to answer this question, as we saw CV benefits appearing after 20 years in those with newly diagnosed type 2 diabetes [28]. However, the patient population in this trial is unique as they are newly diagnosed but already have established CVD (similar to the ORIGIN trial population [29]).

Overall, sitagliptin in TECOS trial came up with the most impressive results (pending FDA reanalysis). We need to keep at the back of our minds the issues related to saxagliptin and alogliptin in some special situations.

## Figures and Tables

**Table 1:** PROactive primary end-point results [4].

	HR (95% CI)
Death	0.96 (0.78–1.18)
Nonfatal MI	0.83 (0.65–1.06)
Stroke	0.81 (0.61–1.07)
Major leg amputation	1.01 (0.58–1.73)
Acute coronary syndrome	0.78 (0.55–1.11)
Coronary revascularization	0.88 (0.72–1.08)
Leg revascularization	1.25 (0.90–1.73)

**Table 2:** PROactive heart failure data [4].

	Pio/placebo (%)	*P* value
Any heart failure report	11/8	<0.0001
Heart failure not requiring hospitalization	5/3	0.003
Heart failure requiring hospitalization	6/4	0.007
Fatal heart failure	1/1	0.634

**Table 3:** Baseline characteristics [6].

Age	57 years or less
Sex	Predominantly males

Average HBA1C	8.2%

Active comparators	(i) Placebo (ii) Metformin (iii) Sulfonylureas (iv) Insulin

**Table 4:** Adverse outcomes [6].

	Odds ratio (95% CI)	*P* value
Myocardial infarction (combined comparator drugs)	1.43 (1.03–1.98)	0.03

Death from CV causes (combined comparator drugs)	1.64 (0.98–2.74)	0.06

**Table 5:** Upper bound 2-sided 95% confidence interval cut-off for approval [7].

≥1.8	Not approvable

1.3–<1.8	Approvable (large CV safety trial required)

<1.3	Approvable (postmarketing CV trial may not be required)

**Table 6:** Saxagliptin polled phase 2b/3 MACE [13].

	Events (%): saxagliptin/placebo	OR (95% CI)
Sponsor MACE	0.5/1	0.5 (0.2–1.2)
Custom MACE (ST)	0.1/0.6	0.21 (0.04–0.8)
Custom MACE (ST + LT)	0.7/1.3	0.52 (0.3–1.0)
SMQ MACE (ST)	1.8/2.0	0.90 (0.6–1.5)
SMQ MACE (ST + LT)	3.1/3.2	0.96 (0.7–1.4)

**Table 7:** 

	SAVOR TIMI 53	EXAMINE	TECOS
Primary composite CV end-points	HR 1.00 [CI: 0.89–1.12] [16]	HR 0.96 [CI: Upper Limit 1.16] [20]	HR 0.98 [CI: 0.88–1.09] [17]

Heart failure	HR 1.27 [CI: 1.07–1.51] [16] FDA reanalysis: HR 1.16 [CI: 1.03–1.30] [22]	HR 1.07 [CI: 0.79–1.46] [20] FDA reanalysis: HR 0.98 [CI: 0.84–1.13] [23]	HR 1.00 [CI: 0.83–1.20] [17] Composite analysis: HR 1.02 [CI: 0.90–1.15] [17]

All-cause mortality (7-day death) MACE: risk	HR 1.23 [CI: 1.02–1.48] [22]	Higher risk of MACE in the following: (i) Smokers, DM duration >10 yrs., metformin nonusers, insulin users, moderate-to-severe renal insufficiency [23]. (ii) Patients from US and Canada [23].	HR 1.01 [CI: 0.90–1.14] [17]

Hypoglycemia	HR 1.16 [CI: 1.08–1.25] [16]	6.7% (A) versus 6.5% (P) [19]	HR 1.12 [CI: 0.89–1.40] SH [17]

## Abbreviations

ACCORD: Action to Control Cardiovascular Risk in Diabetes
ADVANCE: Action in Diabetes and Vascular Disease: Preterax and Diamicron Modified Release Controlled Evaluation
VADT: Veterans Affairs Diabetes Trial
UKPDS: United Kingdom Prospective Diabetes Study
SGLT-2: Sodium Glucose Cotransporter Protein 2
USFDA: United States Food and Drug Administration
SAVOR: Saxagliptin Assessment of Vascular Outcomes Recorded in Patients with Diabetes Mellitus
EXAMINE: Examination of Cardiovascular Outcomes: Alogliptin versus Standard of Care in Patients with Type 2 Diabetes Mellitus and Acute Coronary Syndrome
UGDP: University Group Diabetes Study Program
NYHA: New York Heart Association
CV: Cardiovascular
MI: Myocardial infarction
DPP-4: Dipeptidyl peptidase-4
MACE: Major adverse cardiac events
ST: Short term
LT: Long term
TECOS: The Trial to Evaluate Cardiovascular Outcomes after Treatment with Sitagliptin
HR: Hazard ratio
CI: Confidence interval
NP-Y: Neuropeptide-Y
SP: Substance P
eGFR: Estimated glomerular filtration rate
hHF: Hospitalization due to heart failure
DM: Diabetes mellitus
SH: Severe hypoglycemia
CARMELINA: Cardiovascular Safety and Renal Microvascular Outcome with Linagliptin in Patients with Type 2 Diabetes Mellitus at High Vascular Risk
CAROLINA: Cardiovascular Outcome Study of Linagliptin versus Glimepiride in Early Type 2 Diabetes
ORIGIN: Outcome Reduction with Initial Glargine Intervention.
